# Splenic Rupture: A Rare Complication of Extracorporeal Shock Wave Lithotripsy

**DOI:** 10.5334/jbr-btr.889

**Published:** 2015-12-30

**Authors:** Inneke Willekens, Carola Brussaard, Steven Raeymaeckers, Vincent De Coninck, Johan De Mey

**Affiliations:** 1 Department of Radiology, Universitair Ziekenhuis Brussel (UZ Brussel), and Vrije Universiteit Brussel (VUB), Brussels, Belgium; 2 Department of Urology, Universitair Ziekenhuis Brussel (UZ Brussel), and Vrije Universiteit Brussel (VUB), Brussels, Belgium

## Case report

**Figure 1:**
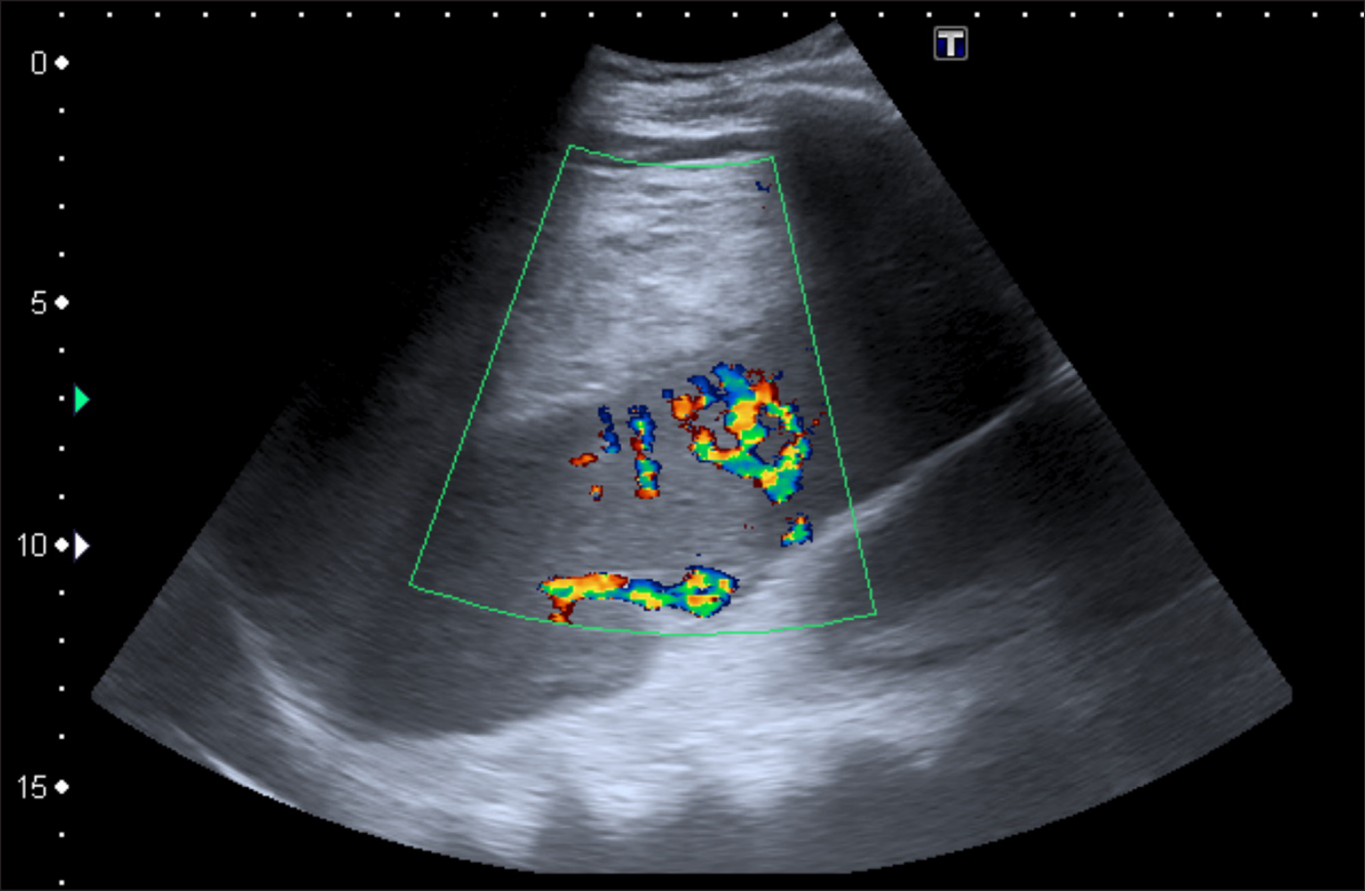
Ultrasound of the spleen with the large subcapsular hyperechoic hematoma.

**Figure 2:**
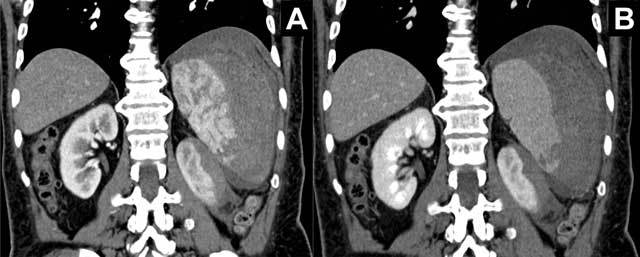
Contrast-enhanced computed tomography with A. Coronal reconstruction in arterial phase and B. Coronal reconstruction in portal venous phase showing the laceration of the lower pole of the spleen with the large subcapsular hematoma. Adjacent to the splenic laceration, a smaller subcapsular hematoma can be seen in the left kidney.

A 41-year-old man presented at the emergency department with complaints of intolerable pain in the left upper quadrant. He had undergone extracorporeal shock wave lithotripsy (ESWL) eight hours prior as treatment for a left-sided kidney stone in the upper pole. Ultrasound demonstrated a large subcapsular hyperechoic collection in the spleen (Figure 1). A computerized tomography (CT) scan confirmed a laceration of the lower pole of the spleen with a subcapsular hematoma and a discrete amount of surrounding free fluid. Adjacent to the splenic laceration, a smaller subcapsular hematoma was also present in the left kidney (Figure 2). In the meantime, a fragmented stone in the proximal left ureter was visualized (1400 HU). The therapy was conservative with hemodynamic follow-up in the intensive care unit with normal patient recovery

## Discussion

Medical imaging plays an important role in the treatment selection for renal stones [1,2]. The choice for ESWL is based on stone size (between 6–16 mm), stone position (in the kidney or proximal ureter) and the stone-to-skin-distance (favorable less than 10 cm). Stones with a high and homogeneous density (greater than1000 HU) are less fragile and less suitable for ESWL. The complications are more frequent and more serious when a high energy level is used. In this patient, the sum of the distance of the several fragments after ESWL was about 15 mm. The distance from the kidney to the skin was less than 10 cm, making a right focusing of the energy bundle possible. The patient was treated with a high energy level (250 J) because of the very high density of the stone (greater than1400 HU). For patients undergoing ESWL who have previously been diagnosed with splenic abnormalities, special care must be taken to avoid collateral damage in adjacent organs, as they are more vulnerable to shearing lesions [1]. Review of the CT scan of this patient reveals a spleen with a longest axis of 15.5 cm, suggesting splenomegaly.

Splenic laceration is a rare complication of abdominal procedures, occurring most often after colonoscopy. It has been described as a complication of ESWL [3]. Extracorporeal shock-wave lithotripsy is a widely used non-invasive treatment method for renal and some ureteric calculi. This procedure is generally considered safe. Serious complications causing ongoing morbidity – such as impaired kidney function with renal bleeding after repeated treatment or even mortality – are rare, affecting less than 1% of patients.

There have been eight previous cases reported of splenic rupture after ESWL. All cases showed patients received more than 2000 shocks at moderate to high energy level and went on to have splenectomy [3]. These cases reflect the more serious end of the spectrum of splenic injury after ESWL. Although splenic rupture is rare, it should be considered in patients presenting with upper-quadrant pain after ESWL. Physical findings associated with splenic injury after ESWL include left upper quadrant or generalized abdominal tenderness, abdominal tenderness, as well as left lower chest wall tenderness. As a result of blood loss in the extravascular space, hypotension and even hemorrhagic shock are possible.

Imaging also plays an important role in the work-out of post-procedural complications. Abdominal ultrasound and CT of the abdomen may reveal a rupture of the spleen. The treatment for splenic rupture depends on its degree and the hemodynamic stability of the patient. Due to the large intracapsular hematoma and his hemodynamic stability, the treatment of our patient was conservative with strict monitoring on the intensive care unit.

As a take-home point, we want to state that the role of the radiologist in the reporting of a CT scan for urolithiasis is not restricted to the description of the number, the size or the density of the stones. As organs move up to 5 cm during ESWL, we must be aware that a lot of energy does not reach the stone, but can damage other organs [1]. Therefore, it is recommendable to warn for organs which might be hit by the energy waves, such as an enlarged spleen, in an attempt to avoid post-procedural complications.

## Competing Interests

The author declares that they have no competing interests.
